# Tree diversity reduces pest damage in mature forests across Europe

**DOI:** 10.1098/rsbl.2015.1037

**Published:** 2016-04

**Authors:** Virginie Guyot, Bastien Castagneyrol, Aude Vialatte, Marc Deconchat, Hervé Jactel

**Affiliations:** 1 INRA, DYNAFOR, UMR 1201, 31326 Castanet-Tolosan, France; 2 INPT-ENSAT, DYNAFOR, Université de Toulouse, UMR 1201, 31326 Castanet-Tolosan, France; 3 INRA, BIOGECO, Université de Bordeaux, 33610 Cestas, France

**Keywords:** associational resistance, biodiversity, ecosystem functioning

## Abstract

Forest pest damage is expected to increase with global change. Tree diversity could mitigate this impact, but unambiguous demonstration of the diversity–resistance relationship is lacking in semi-natural mature forests. We used a network of 208 forest plots sampled along two orthogonal gradients of increasing tree species richness and latitudes to assess total tree defoliation in Europe. We found a positive relationship between tree species richness and resistance to insect herbivores: overall damage to broadleaved species significantly decreased with the number of tree species in mature forests. This pattern of associational resistance was frequently observed across tree species and countries, irrespective of their climate. These findings confirm the greater potential of mixed forests to face future biotic disturbances in a changing world.

## 1. Introduction

Biodiversity is widely acknowledged to support many forest ecosystem functions [1] and services [2]. However, they can be jeopardized by pest damage [3], which are likely to increase under global change, including climate change [4] and biological invasions [5]. Preventive pest management methods are therefore urgently needed to preserve the integrity and functioning of forests.

Regarding the pest regulation service, empirical studies and quantitative reviews have shown that diverse forests are less prone to pest insects than tree monocultures [6,7] including to invasive species [8], which suggests associational resistance (AR [9]). Main mechanisms underlying AR include resource dilution, reduced host apparency and impact of natural enemies [6–9]. However, recent studies also reported the opposite, i.e. more damage in mixed forests (associational susceptibility, AS [10]), or simply no effect of diversity [11]. Still, these results mainly rely on studies that assessed damage on young trees in tree diversity experiments. We are therefore lacking an estimate of forest diversity effect on overall tree damage in semi-natural mature stands.

Moreover, insect herbivory changes along biogeographic gradients. Plant–herbivore–predator interactions are clearly dependent on temperatures and precipitations [12]. Whether this affects the persistence of AR across a large range of latitudes and thus whether AR could stand under warmer climates is still unknown.

We estimated crown defoliation in semi-natural mature forests sampled along two orthogonal gradients of increasing tree species richness and latitudes in Europe [13]. By controlling these two factors, we could investigate the stationarity of AR patterns across a large range of climatic conditions. More specifically, we compared the effects of forest diversity on total defoliation at both the stand and the tree species levels.

## 2. Material and methods

Insect damage was assessed in a network of 208 semi-natural mature forests in six European regions of Mediterranean, temperate and boreal areas [13]. In each region, forest plots were sampled under homogeneous abiotic and management conditions, along a gradient of tree diversity ranging from monocultures of the locally most common tree species (‘focal species') to mixtures of two, three, four or five species, depending on the regional species pool. Each plot was delimited by a square of 900 m^2^ surrounded by a 10 m buffer area to avoid edge effects. A total of 11 broadleaved and four conifer focal species were assessed (table 1). Six individual trees per focal species were sampled at random among the dominant ones in pure stands, three in mixed stands.

**Table 1.** List of focal tree species assessed for insect defoliation along gradients of tree species richness in six European regions.

	characteristics of sampled forests in the six European regions						
	*Colline Metalifere* (Italy)	*Alto Tajo* (Spain)	*Hainich* (Germany)	*Bialowieza* (Poland)	*Râsca* (Romania)	*North Karelia* (Finland)	
species richness levels	1–4	1–4	1–4	1–5	1–4	1–3	
plots per richness level	9/10/9/7	11/18/4/3	6/14/14/4	6/11/13/11/2	8/10/8/2	11/14/3	
mean forest age (years)	62	90	111	92	85	42	
mean temperature (°C)	13.1	9.7	7.4	6.9	5.5	2.1	
mean precipitation (mm)	726	534	696	581	692	633	

focal tree species	number of sampled trees per species per region						mean (±s.e.) % defoliation per plot
*Carpinus betulus*				82			14.1 (±1.6)
*Castanea sativa*	73						13.4 (±1.6)
*Quercus robur - petraea*	57		45	75			11.6 (±1.4)
*Quercus faginea*		77					9.2 (±1.0)
*Fagus sylvatica*			94		65		6.7 (±0.7)
*Fraxinus excelsior*			71				5.9 (±0.7)
*Acer pseudoplatanus*			53		43		5.3 (±0.4)
*Quercus cerris*	74						4.9 (±0.6)
*Betula pendula*				72		62	3.8 (±0.2)
*Quercus ilex*	74	51					2.3 (±0.3)
*Ostrya carpinifolia*	58						1.7 (±0.3)
*Picea abies*			34	75	53	61	0.9 (±0.2)
*Pinus nigra*		76					0.5 (±0.2)
*Abies alba*					52		0.5 (±0.2)
*Pinus sylvestris*		56		75		61	0.1 (±0.0)

Total insect damage in sampled trees was estimated using the crown condition survey protocol developed by Guyot *et al.* [8] according to the ICP Forest manual [14]. We considered damage as leaf area reduction in tree crown, hereafter termed as defoliation. To assess defoliation, a comparison was made between the focal tree and a ‘reference tree’, i.e. a healthy conspecific tree of similar age, leaf phenology and environmental conditions in its vicinity. The assessment was done with binoculars by the same observer (V.G.) for all trees, from at least two sides (more if visibility was limited) of the crown to account for all damage. Where different percentages of defoliation were attributed to a focal tree from different sides, the mean percentage was used. To confirm that crown defoliation was owing to insect damage, herbivory was assessed on a leaf sample collected on each studied tree (electronic supplementary material, S1). Three regions were visited in summer 2012 and the other three in summer 2013, starting from the south to follow leaf phenology, but all trees from a given region were sampled within the same three weeks.

The mean percentage of defoliation per plot and per species was used as response variable (after log transformation). Because defoliation of conifers was very low (on average less than 1%) and poorly reliable, it was not considered as response variable in our analyses. Plots including conifers were, however, retained in analyses such that presence of conifers was accounted for in explanatory variables. We focused on defoliation of 11 broadleaved species, which was assessed in broadleaved monocultures, broadleaved–broadleaved mixtures or broadleaved–conifer mixtures.

First, linear-mixed effect models were used (*lmer* function in the *lmerTest* package in R [15,16]) to test the effect of tree species richness, mean annual temperature and precipitation of the region, and all interactions on defoliation. Explanatory variables were scaled and centred to allow comparison of model parameters. We applied model simplification with backward elimination of effects, according to the principle of marginality (*step* function). Model parameters were estimated with the final model. Significance of explanatory variables was tested using type III sum of squares. Coefficients of determination *R*^2^ [17] were calculated with *r.squaredGLMM* function in the *MuMIn* package in R [18].

The effect of tree species richness on species-specific defoliation was modelled for each broadleaved species in each region separately using linear models in a meta-analytical approach (electronic supplementary material, S1).

## 3. Results

Among the 1669 sampled trees, crown defoliation varied from 0 to 62.5%. At the plot level, the mean crown defoliation ranged from 2 to 14% in broadleaved species (crown defoliation averaged 7.2 ± 1.1%, whereas leaf herbivory was 7.2 ± 1.3%) and was consistently less than 1% in conifers (table 1).

**Figure 1.**
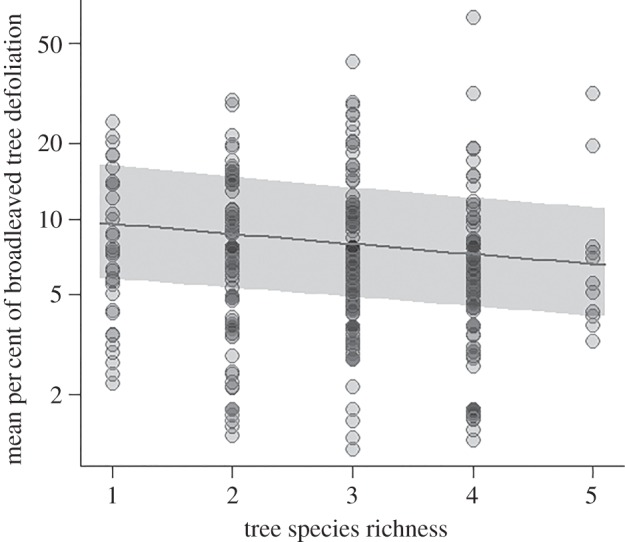
Relationship between mean percentage of broadleaved tree defoliation per forest plot (*n* = 328) and tree species richness in semi-natural, mature European forests. The solid line and the shaded area show predictions from linear-mixed model with corresponding confidence interval.

At the plot level, defoliation decreased significantly with tree species richness (*F* = 16.01, *p* < 0.001, figure 1). Predicted mean tree defoliation in broadleaves varied from 9.6% in monocultures to 6.6% in mixtures of five species. The effect of tree species richness on defoliation was independent of temperature (interaction: *F* = 1.18, *p* = 0.278) and precipitation of the region (interaction: *F* = 1.2, *p* = 0.267). Simple effects of temperature (*F* = 0.03, *p* = 0.858) and precipitation (*F* = 0.01, *p* = 0.911) were not significant. Variance explained by the fixed effects (species richness, 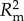) was low compared with random effects (region and species identity, 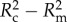, table 2).

**Table 2.** Values of explained variance (coefficients of determination, *R*^2^) and model estimates of linear-mixed models used for testing the effect of tree species richness on mean defoliation in broadleaved species accounting for all regions or each of them. Marginal 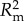 represents the variance explained by fixed factors, whereas conditional 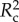 is interpreted as variance explained by both fixed and random factors (i.e. the entire model) [18]. Models with significant *P*-values are in italics.

region	estimate	±s.e.	*F*-value	*P*-value	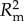	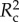
all	*−0.11*	*0.03*	*16.01*	*<0.001*	*0.01*	*0.77*
Italy	−0.12	0.05	6.51	*0.013*	0.02	0.70
Spain	−0.27	0.07	16.73	*<0.001*	0.06	0.88
Germany	−0.05	0.06	0.79	0.378	0.01	0.45
Poland	−0.08	0.06	1.82	0.182	0.01	0.65
Romania	−0.09	0.05	2.89	0.100	0.04	0.54
Finland^a^	−0.01	0.08	0.01	0.932	0	0

^a^As there was only one broadleaved species in Finland, linear model was used for this region.

**Figure 2.**
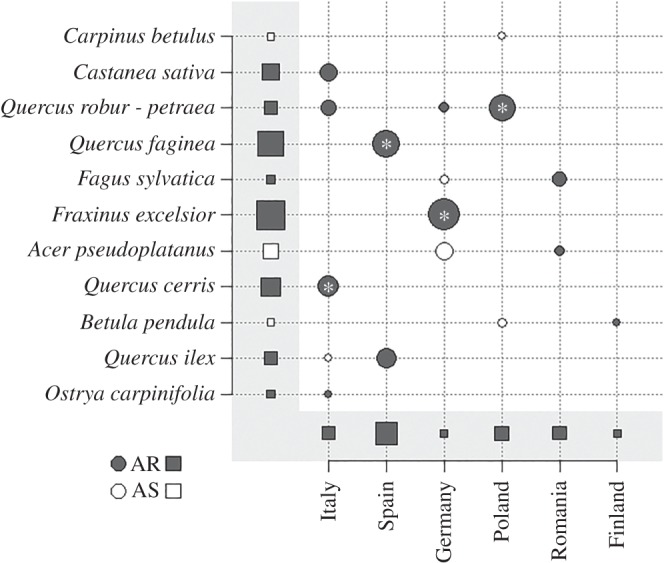
Species-specific and country-specific responses of defoliation to tree species richness. Symbol size is proportional to model parameter estimate (i.e. regression slope). Dark dots indicate negative slopes (associational resistance, AR), white dots indicate positive slopes (associational susceptibility, AS). An asterisk within a dot indicates a significant relationship. Within grey areas, squares represent weighted mean of slopes across species and across countries. Countries were ordered from the warmest to the coldest.

At the tree species level, AR was the most common pattern (table 2 and figure 2). It was observed in all six regions (and confirmed by region-specific models, electronic supplementary material, figure S2) and in eight out of 11 broadleaved species, although this effect was significant in only four species × country combinations. None of the few tendencies of AS was significant.

## 4. Discussion

Based on a network of more than 200 plots along two explicit orthogonal gradients of tree species richness and latitudes [13] our study demonstrates an overall positive relationship between tree species richness and resistance of broadleaved species to insect defoliators. Although a large part of crown defoliation variability remained unexplained, this pattern was consistent across several broadleaved species and all regions, irrespective of their climate. This is the first demonstration of large-scale AR in semi-natural mature forests.

As for agricultural crops [19], previous meta-analyses reported reduced insect herbivory in more diverse forests [6,7] but they mainly focused on damage made by one particular pest insect on a given tree species grown as pure versus mixed stands. They failed to address the effect of diversity on total insect damage, which may be more relevant to predicting their impact on tree growth and ecosystem functioning. Here, we found that overall resistance of broadleaved species to herbivory was higher in mixed stands than in pure stands, regardless of the damaging agent. It is noteworthy that tree productivity increased with tree species richness across the same plot network [20].

The AR paradigm was questioned by recent studies showing no [9] or opposite [10] patterns of diversity–resistance relationships. However, they were both conducted in rather small-scale tree diversity experiments and not in semi-natural managed forests. In addition, these studies dealt with young trees (less than 15 years), whereas we assessed damage on mature trees (more than 40 years). It was already noted that the effects of tree species diversity on insect herbivory is more pronounced in older trees [21], which may be owing to two, non-exclusive, mechanisms: (i) foliar defences against herbivores accumulate and change in composition with tree ontogeny [22], whereas heterospecific neighbours can affect these leaf traits [23]; (ii) forests recruit an increasing number of specialist herbivores as they are ageing [24], whereas the magnitude of AR is known to be higher against mono- and oligophagous herbivores [6,7].

The study was not designed to allow the investigation of AR mechanisms. However, tree species richness was retained as the best explanatory variable of broadleaved defoliation that is compatible with the two main ecological processes at work: bottom-up effects of plant–plant interactions and top-down effects involving natural enemies [6,9]. Host tree concentration, frequency or apparency [25] are likely to be reduced in the presence of an increasing number of non-host tree species (i.e. bottom-up processes). More diverse forests should shelter more parasitoids or predators and provide them with more abundant and diverse complementarity in feed and nesting resources (i.e. top-down processes).

The main limitation of the study is that we could not assess insect herbivory under pest outbreak conditions (the rate of defoliation on broadleaves was on average close to 10%, but see [8]), neither could we include conifers that were virtually undamaged. It will be therefore of interest to further challenge the AR hypothesis and underlying ecological mechanisms in more stringent conditions, for example during outbreaks of conifer bark beetle. Future studies should also investigate factors accounting for unexplained variance in tree defoliation at the different regions (table 2) such as landscape heterogeneity and composition of local species pools of insect herbivores.

## Supplementary Material

FunDiv-resistance-SM1

## Supplementary Material

FunDiv-resistance-SM2

## Data accessibility

Electronic supplementary material, table S2 reports all data used for statistical analyses.
