# Supplementary material for: Tree diversity reduces pest damage in mature forests across Europe
Source: Biol Lett. 2016 Apr;12(4):20151037. doi: 10.1098/rsbl.2015.1037 (PMC4881340; doi:10.1098/rsbl.2015.1037)
Supplement: FunDiv-resistance-SM1 [file rsbl20151037supp1.docx]

**Tree diversity reduces pest damage in mature forests across Europe**

Virginie Guyot^1,3^, Bastien Castagneyrol^3^, Aude Vialatte^1,2^, Marc Deconchat^1^, Hervé Jactel^3^

^1^ INRA, DYNAFOR, UMR 1201, 31326 Castanet Tolosan, France

^2^ Université de Toulouse, INPT-ENSAT, DYNAFOR, UMR 1201, 31326 Castanet Tolosan, France

^3^ BIOGECO, INRA, Univ. Bordeaux, 33610 Cestas, France

**Supplementary material**

**Assessment of defoliation on leaves**

To confirm that crown defoliation on each sampled broadleaved tree was mostly due to insect herbivory, a leaf sample was collected at the same time as crown assessment. Two light exposed branches were cut by tree climbers, one at the top and the other in the middle of the crown. Thirty leaves per branch were collected at random and frozen at -18°C until assessment. Leaf area removed (*LAR*) by chewers was visually estimated [Johnson et al. 2016] as chewing damage are the most detectable during crown evaluation. The average of *LAR* estimated on leaf sample was then calculated for each sampled tree and then aggregated at the plot level per broadleaved species, per region.

**Correlation of defoliation assessed on tree crown and leaf sample**

Total crown defoliation of broadleaved species was correlated to chewers damage assessed on leaves (*n* = 328, Pearson’s *r* = 0.57, *P* < 0.001, Fig.S1).


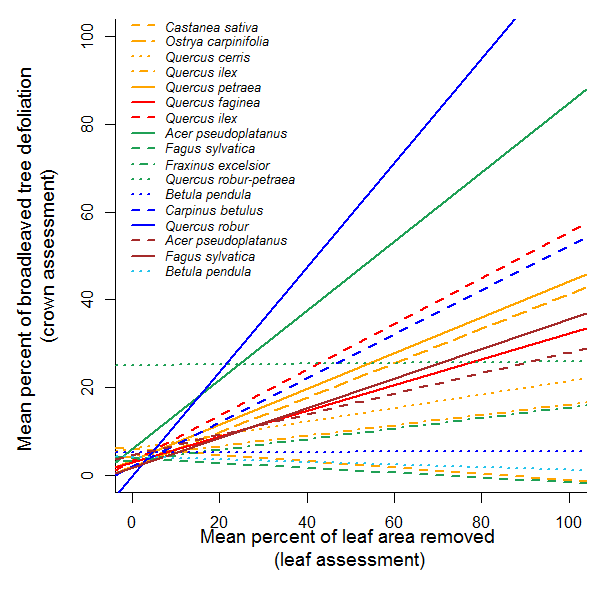


**Fig.S1** Relationship between total crown defoliation and mean leaf area removed by chewers on leaves, collected in the same trees of 11 broadleaved species. Each line represents mean percent of damage estimated per broadleaved species and per studied region (orange = Italy, red = Spain, green = Germany, blue = Poland, brown = Romania, cyan = Finland).

**Method for averaging slopes using weighted means (meta-analytical approach)**

We used a meta-analytical approach [Koricheva et al. 2013] to combine country-specific and species-specific responses to tree species richness, by aggregating model parameter estimates (i.e. regression slopes) across regions for a given tree species, or across species for a given region. Defoliations were scaled and centered before estimating regression slope and standard error [Schielzeth 2010]. We calculated combined weighted means, using standard error around country- and species-specific slopes as a weighting factor (eqn(2)) [Koricheva et al. 2013].

$\text{b}=\frac{\sum_{\text{i}=1}^{\text{k}} \text{w}_{\text{i}}\text{b}_{\text{i}}}{\sum_{\text{i}=1}^{\text{k}} \text{w}_{\text{i}}}$ eqn(2)

with *b_i_* the slope of the species richness - crown defoliation relationship for a given focal tree species in a given region and *w_i_ = 1/v_i_* with *vi* the variance of the slope *b_i_* [Becker et al. 2007].


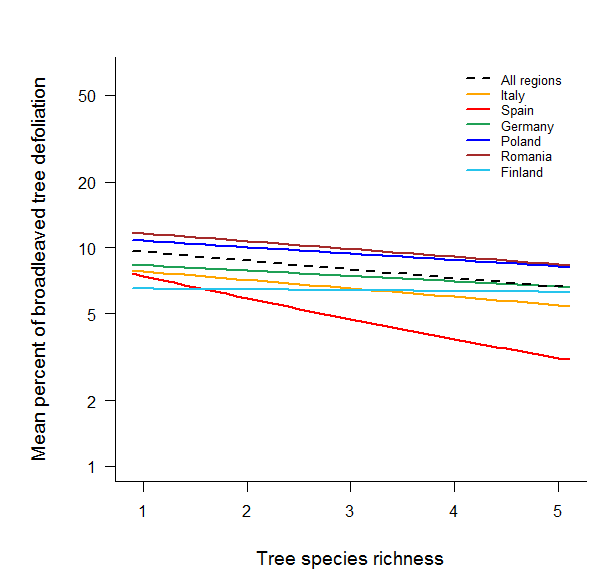


**Fig.S2** Relationships between mean percent of broadleaved tree defoliation per plot and tree species richness in mature forests of six European regions.

The solid lines show predictions from linear mixed model used for all and each region, excepted for Finland where predictions are from a linear model.

**References**

Becker BJ, Wu MJ. 2007 The synthesis of regression slopes in meta-analysis. *Stat. Sci.* **22**, 414-429. (doi:10.1214/07-STS243)

Koricheva J, Gurevitch J, Mengersen K. 2013 Handbook of meta-analysis in ecology and evolution. Princeton University Press.

Schielzeth H. 2010 Simple means to improve the interpretability of regression coefficients. *Meth. Ecol. Evol.* **1**, 103-113. (doi:10.1111/j.2041-210X.2010.00012.x)

Johnson MTJ, Bertrand JA, Turcotte MM. 2016 Precision and accuracy in quantifying herbivory, *Ecological Entomology* **41** 112-121. (doi:10.1111/een.12280)
