# Supplementary material for: Tree diversity reduces pest damage in mature forests across Europe
Source: Biol Lett. 2016 Apr;12(4):20151037. doi: 10.1098/rsbl.2015.1037 (PMC4881340; doi:10.1098/rsbl.2015.1037)
Supplement: FunDiv-resistance-SM2 [file rsbl20151037supp2.pdf]

Tree diversity reduces pest damage in mature forests across Europe

Virginie Guyot[1,3], Bastien Castagneyrol[3], Aude Vialatte[1,2], Marc Deconchat[1], Hervé Jactel[3]

[1] INRA, DYNAFOR, UMR 1201, 31326 Castanet Tolosan, France

[2] Université de Toulouse, INPT-ENSAT, DYNAFOR, UMR 1201, 31326 Castanet Tolosan, France

[3] BIOGECO, INRA, Univ. Bordeaux, 33610 Cestas, France

Supplementary material (data sharing)

Table S2 Mean percent of defoliation per plot and per species in each studied region with the four diversity variables (tree species richness ("richness"), Shannon index of tree diversity ("shannon"), proportion of focal species ("focal.prop"), proportion of conifers ("conifer.prop")) and mean annual temperature ("temperature").

country	id.plot	species	defoliation	richness	shannon	focal.prop	conifer.prop	temperature
Finland	FIN01	PICABI	0.0	2	0.60	76.5	98.7	2.6
Finland	FIN01	PINSYL	0.4	2	0.60	22.2	98.7	2.6
Finland	FIN02	BETPEN	5.7	2	0.75	74.3	25.7	2.4
Finland	FIN02	PICABI	0.0	2	0.75	25.7	25.7	2.4
Finland	FIN03	BETPEN	5.2	1	1.02	83.5	16.5	2.4
Finland	FIN04	BETPEN	5.3	2	0.90	36.0	64.0	2.5
Finland	FIN04	PICABI	0.2	2	0.90	64.0	64.0	2.5
Finland	FIN05	BETPEN	3.9	2	0.63	24.9	75.1	2.6
Finland	FIN05	PINSYL	0.0	2	0.63	73.6	75.1	2.6
Finland	FIN06	PICABI	0.0	1	0.04	99.4	99.4	2.5
Finland	FIN07	BETPEN	4.8	1	0.04	99.7	0.3	2.3
Finland	FIN08	BETPEN	3.2	1	0.05	99.0	1.0	2.3
Finland	FIN09	PINSYL	0.0	1	0.08	98.6	99.1	2.2
Finland	FIN10	PICABI	2.9	1	0.07	98.8	98.8	2.1
Finland	FIN11	BETPEN	4.6	1	0.12	97.5	2.5	2.2
Finland	FIN12	BETPEN	3.6	3	1.04	25.8	74.2	1.4
Finland	FIN12	PICABI	0.2	3	1.04	11.8	74.2	1.4
Finland	FIN12	PINSYL	0.0	3	1.04	62.4	74.2	1.4
Finland	FIN13	BETPEN	1.1	2	0.82	20.9	79.1	1.4
Finland	FIN13	PINSYL	0.2	2	0.82	74.3	79.1	1.4
Finland	FIN14	PINSYL	0.1	1	0.00	100.0	100.0	1.8
Finland	FIN15	BETPEN	5.0	2	0.59	23.7	76.3	1.9
Finland	FIN15	PINSYL	0.0	2	0.59	75.6	76.3	1.9
Finland	FIN16	PINSYL	0.9	1	0.15	97.0	97.9	1.8
Finland	FIN17	PICABI	0.2	2	0.55	23.5	100.0	1.8
Finland	FIN17	PINSYL	0.0	2	0.55	76.5	100.0	1.8
Finland	FIN18	PICABI	0.0	1	0.05	99.2	99.2	2.0
Finland	FIN19	PICABI	0.0	2	0.71	46.9	99.8	2.1
Finland	FIN19	PINSYL	0.0	2	0.71	52.9	99.8	2.1
Finland	FIN20	BETPEN	6.8	2	0.50	16.8	83.2	2.2
Finland	FIN20	PICABI	1.9	2	0.50	83.2	83.2	2.2
Finland	FIN21	PINSYL	0.0	1	0.04	99.2	100.0	2.0
Finland	FIN22	PICABI	0.0	2	0.69	54.8	100.0	2.1
Finland	FIN22	PINSYL	0.0	2	0.69	45.2	100.0	2.1
Finland	FIN23	PICABI	0.2	1	0.17	95.9	100.0	2.1
Finland	FIN24	BETPEN	6.8	2	0.78	26.0	74.0	1.9
Finland	FIN24	PICABI	0.0	2	0.78	72.7	74.0	1.9
Finland	FIN25	BETPEN	3.4	3	0.98	13.1	86.9	2.1
Finland	FIN25	PICABI	0.0	3	0.98	58.9	86.9	2.1
Finland	FIN25	PINSYL	0.0	3	0.98	27.9	86.9	2.1
Finland	FIN26	BETPEN	4.1	3	1.26	29.9	70.1	2.1

country	id.plot	species	defoliation	richness	shannon	focal.prop	conifer.prop	temperature
Finland	FIN26	PICABI	0.0	3	1.26	40.2	70.1	2.1
Finland	FIN26	PINSYL	0.0	3	1.26	29.9	70.1	2.1
Finland	FIN27	BETPEN	6.8	3	0.85	8.8	91.2	2.5
Finland	FIN27	PICABI	0.0	3	0.85	25.3	91.2	2.5
Finland	FIN27	PINSYL	0.0	3	0.85	65.9	91.2	2.5
Finland	FIN28	BETPEN	4.3	2	1.04	48.8	51.2	2.5
Finland	FIN28	PINSYL	0.0	2	1.04	49.8	51.2	2.5
Germany	GER01	FAGSYL	3.3	1	0.00	100.0	0.0	7.3
Germany	GER02	FAGSYL	4.1	1	0.00	100.0	0.0	7.4
Germany	GER03	ACEPSE	6.8	2	0.52	10.4	3.0	7.8
Germany	GER03	FRAEXC	7.9	2	0.52	85.6	3.0	7.8
Germany	GER04	FRAEXC	9.0	1	0.36	91.0	0.0	7.9
Germany	GER05	QUERCUS	13.0	1	0.45	87.9	0.0	7.8
Germany	GER06	ACEPSE	1.2	2	0.69	5.0	0.0	7.8
Germany	GER06	FAGSYL	1.8	2	0.69	20.0	0.0	7.8
Germany	GER06	QUERCUS	8.3	2	0.69	75.1	0.0	7.8
Germany	GER07	PICABI	5.8	1	0.26	92.7	92.7	6.9
Germany	GER08	PICABI	0.3	1	0.24	94.2	94.2	7.8
Germany	GER09	ACEPSE	8.4	3	1.05	42.6	0.0	7.4
Germany	GER09	FAGSYL	3.5	3	1.05	38.0	0.0	7.4
Germany	GER09	FRAEXC	4.2	3	1.05	19.5	0.0	7.4
Germany	GER10	ACEPSE	3.2	3	1.08	38.2	0.0	7.4
Germany	GER10	FAGSYL	3.6	3	1.08	37.8	0.0	7.4
Germany	GER10	FRAEXC	2.8	3	1.08	24.0	0.0	7.4
Germany	GER11	FAGSYL	3.1	2	0.86	43.8	0.0	7.1
Germany	GER11	FRAEXC	7.7	2	0.86	51.0	0.0	7.1
Germany	GER12	FAGSYL	5.7	2	0.83	54.1	0.0	7.3
Germany	GER12	QUERCUS	28.9	2	0.83	41.8	0.0	7.3
Germany	GER13	ACEPSE	2.3	3	1.03	11.6	0.0	8.0
Germany	GER13	FRAEXC	5.4	3	1.03	29.1	0.0	8.0
Germany	GER13	QUERCUS	6.5	3	1.03	56.6	0.0	8.0
Germany	GER14	FAGSYL	2.8	2	0.91	28.3	63.2	7.8
Germany	GER14	PICABI	1.8	2	0.91	63.2	63.2	7.8
Germany	GER15	FRAEXC	8.1	2	0.61	30.0	70.0	7.4
Germany	GER15	PICABI	0.5	2	0.61	70.0	70.0	7.4
Germany	GER16	FAGSYL	2.8	3	1.00	14.6	47.3	7.6
Germany	GER16	PICABI	2.9	3	1.00	47.3	47.3	7.6
Germany	GER16	QUERCUS	8.3	3	1.00	38.2	47.3	7.6
Germany	GER17	ACEPSE	0.8	2	0.52	21.4	0.0	7.5
Germany	GER17	FAGSYL	0.8	2	0.52	78.6	0.0	7.5
Germany	GER18	ACEPSE	8.8	3	0.83	23.3	1.9	6.9
Germany	GER18	FAGSYL	3.8	3	0.83	5.8	1.9	6.9
Germany	GER18	FRAEXC	2.6	3	0.83	69.0	1.9	6.9
Germany	GER19	ACEPSE	9.6	3	0.77	16.9	0.0	7.3
Germany	GER19	FAGSYL	2.8	3	0.77	10.7	0.0	7.3

country	id.plot	species	defoliation	richness	shannon	focal.prop	conifer.prop	temperature
Germany	GER19	QUERCUS	8.8	3	0.77	72.4	0.0	7.3
Germany	GER20	FAGSYL	1.8	3	1.03	22.3	0.0	7.7
Germany	GER20	FRAEXC	6.8	3	1.03	26.2	0.0	7.7
Germany	GER20	QUERCUS	19.1	3	1.03	51.6	0.0	7.7
Germany	GER21	ACEPSE	6.8	4	1.07	14.3	0.0	7.3
Germany	GER21	FAGSYL	4.8	4	1.07	62.6	0.0	7.3
Germany	GER21	QUERCUS	62.5	4	1.07	12.6	0.0	7.3
Germany	GER22	FAGSYL	5.3	2	0.93	55.8	4.5	7.4
Germany	GER22	FRAEXC	6.1	2	0.93	37.3	4.5	7.4
Germany	GER23	FAGSYL	5.2	3	1.02	52.0	26.1	7.5
Germany	GER23	PICABI	0.2	3	1.02	26.1	26.1	7.5
Germany	GER23	QUERCUS	10.1	3	1.02	21.9	26.1	7.5
Germany	GER24	ACEPSE	4.4	3	1.04	35.8	0.0	7.2
Germany	GER24	FAGSYL	3.2	3	1.04	45.0	0.0	7.2
Germany	GER24	FRAEXC	5.8	3	1.04	19.2	0.0	7.2
Germany	GER25	ACEPSE	5.5	3	1.05	19.6	0.0	7.5
Germany	GER25	FAGSYL	6.0	3	1.05	39.4	0.0	7.5
Germany	GER25	QUERCUS	27.4	3	1.05	41.0	0.0	7.5
Germany	GER26	ACEPSE	4.2	3	1.11	50.4	4.0	7.0
Germany	GER26	FAGSYL	2.8	3	1.11	31.9	4.0	7.0
Germany	GER26	FRAEXC	6.8	3	1.11	13.6	4.0	7.0
Germany	GER27	ACEPSE	2.8	4	1.22	11.7	0.0	7.0
Germany	GER27	FAGSYL	0.8	4	1.22	16.4	0.0	7.0
Germany	GER27	FRAEXC	1.8	4	1.22	51.0	0.0	7.0
Germany	GER27	QUERCUS	10.8	4	1.22	21.0	0.0	7.0
Germany	GER28	ACEPSE	1.2	2	0.69	51.7	0.0	8.0
Germany	GER28	FRAEXC	6.8	2	0.69	48.3	0.0	8.0
Germany	GER29	FAGSYL	10.4	3	1.15	56.5	3.3	7.3
Germany	GER29	FRAEXC	2.7	3	1.15	22.8	3.3	7.3
Germany	GER29	QUERCUS	15.5	3	1.15	15.0	3.3	7.3
Germany	GER30	FAGSYL	2.2	3	0.84	65.2	0.0	7.2
Germany	GER30	FRAEXC	4.5	3	0.84	8.2	0.0	7.2
Germany	GER30	QUERCUS	6.8	3	0.84	26.6	0.0	7.2
Germany	GER31	ACEPSE	0.8	4	1.05	8.0	0.0	6.9
Germany	GER31	FAGSYL	6.8	4	1.05	61.2	0.0	6.9
Germany	GER31	FRAEXC	0.8	4	1.05	21.5	0.0	6.9
Germany	GER31	QUERCUS	2.8	4	1.05	9.4	0.0	6.9
Germany	GER32	ACEPSE	8.4	3	1.07	23.2	0.0	7.1
Germany	GER32	FAGSYL	6.8	3	1.07	40.3	0.0	7.1
Germany	GER32	FRAEXC	5.8	3	1.07	36.5	0.0	7.1
Germany	GER33	ACEPSE	5.4	4	1.34	20.1	15.5	7.5
Germany	GER33	FAGSYL	5.1	4	1.34	33.3	15.5	7.5
Germany	GER33	FRAEXC	4.7	4	1.34	31.2	15.5	7.5
Germany	GER33	PICABI	0.3	4	1.34	15.5	15.5	7.5
Germany	GER34	FAGSYL	2.8	3	0.87	9.4	13.0	7.4

country	id.plot	species	defoliation	richness	shannon	focal.prop	conifer.prop	temperature
Germany	GER34	FRAEXC	14.8	3	0.87	72.7	13.0	7.4
Germany	GER34	PICABI	0.3	3	0.87	13.0	13.0	7.4
Germany	GER35	ACEPSE	3.4	4	1.12	7.5	7.8	7.8
Germany	GER35	FAGSYL	2.0	4	1.12	40.2	7.8	7.8
Germany	GER35	PICABI	9.1	4	1.12	7.8	7.8	7.8
Germany	GER35	QUERCUS	7.2	4	1.12	44.5	7.8	7.8
Germany	GER36	FRAEXC	13.3	2	0.79	68.5	24.1	7.7
Germany	GER36	PICABI	4.5	2	0.79	24.1	24.1	7.7
Germany	GER37	ACEPSE	4.9	4	1.48	20.5	4.0	7.2
Germany	GER37	FAGSYL	4.4	4	1.48	30.2	4.0	7.2
Germany	GER37	FRAEXC	6.0	4	1.48	26.7	4.0	7.2
Germany	GER37	QUERCUS	16.1	4	1.48	18.6	4.0	7.2
Germany	GER38	ACEPSE	4.0	3	1.12	13.6	0.8	6.9
Germany	GER38	FAGSYL	2.5	3	1.12	55.7	0.8	6.9
Germany	GER38	FRAEXC	1.8	3	1.12	25.5	0.8	6.9
Italy	ITA01	QUECER	5.7	2	0.72	38.7	0.0	7.0
Italy	ITA01	QUERCUS	9.2	2	0.72	60.2	0.0	7.0
Italy	ITA02	OSTCAR	0.7	4	1.37	18.5	0.0	13.6
Italy	ITA02	QUECER	4.1	4	1.37	27.6	0.0	13.6
Italy	ITA02	QUEILE	2.5	4	1.37	25.2	0.0	13.6
Italy	ITA02	QUERCUS	6.8	4	1.37	28.8	0.0	13.6
Italy	ITA03	OSTCAR	4.5	1	0.41	85.9	0.0	13.4
Italy	ITA04	QUERCUS	7.6	1	0.42	89.5	0.0	13.6
Italy	ITA05	OSTCAR	1.8	3	1.19	29.1	0.0	13.6
Italy	ITA05	QUEILE	6.5	3	1.19	14.3	0.0	13.6
Italy	ITA05	QUERCUS	6.8	3	1.19	48.4	0.0	13.6
Italy	ITA06	QUEILE	1.9	1	0.00	100.0	0.0	13.7
Italy	ITA07	CASSAT	23.5	1	0.00	100.0	0.0	13.2
Italy	ITA08	OSTCAR	0.7	2	0.59	72.6	0.0	13.2
Italy	ITA08	QUEILE	3.9	2	0.59	27.4	0.0	13.2
Italy	ITA09	OSTCAR	0.6	3	1.10	16.1	0.0	13.4
Italy	ITA09	QUECER	6.3	3	1.10	23.3	0.0	13.4
Italy	ITA09	QUEILE	2.2	3	1.10	56.0	0.0	13.4
Italy	ITA10	CASSAT	15.2	3	1.02	20.6	0.0	13.0
Italy	ITA10	QUEILE	4.3	3	1.02	52.1	0.0	13.0
Italy	ITA10	QUERCUS	6.8	3	1.02	27.3	0.0	13.0
Italy	ITA11	CASSAT	7.3	4	1.21	50.6	0.0	14.0
Italy	ITA11	OSTCAR	3.4	4	1.21	23.2	0.0	14.0
Italy	ITA11	QUEILE	6.3	4	1.21	15.7	0.0	14.0
Italy	ITA11	QUERCUS	5.4	4	1.21	10.5	0.0	14.0
Italy	ITA12	CASSAT	17.1	1	0.03	99.5	0.0	13.0
Italy	ITA13	CASSAT	9.8	2	0.76	32.1	0.0	13.0
Italy	ITA13	QUECER	6.8	2	0.76	65.1	0.0	13.0
Italy	ITA14	CASSAT	10.7	3	1.06	46.2	0.0	13.5
Italy	ITA14	QUECER	11.4	3	1.06	27.1	0.0	13.5

country	id.plot	species	defoliation	richness	shannon	focal.prop	conifer.prop	temperature
Italy	ITA14	QUEILE	4.2	3	1.06	26.7	0.0	13.5
Italy	ITA15	QUECER	5.3	1	0.00	100.0	0.0	13.5
Italy	ITA16	QUEILE	1.4	1	0.11	97.8	0.0	13.4
Italy	ITA17	QUEILE	3.9	2	0.86	62.9	0.0	13.6
Italy	ITA17	QUERCUS	6.8	2	0.86	28.3	0.0	13.6
Italy	ITA18	OSTCAR	1.6	4	1.35	19.3	0.0	13.6
Italy	ITA18	QUECER	3.5	4	1.35	17.7	0.0	13.6
Italy	ITA18	QUEILE	2.1	4	1.35	35.1	0.0	13.6
Italy	ITA18	QUERCUS	6.8	4	1.35	27.9	0.0	13.6
Italy	ITA19	OSTCAR	2.5	2	0.70	23.4	0.0	13.3
Italy	ITA19	QUECER	3.8	2	0.70	72.6	0.0	13.3
Italy	ITA20	QUECER	3.4	3	0.94	60.5	0.0	13.3
Italy	ITA20	QUEILE	2.8	3	0.94	20.0	0.0	13.3
Italy	ITA20	QUERCUS	6.8	3	0.94	19.5	0.0	13.3
Italy	ITA21	CASSAT	16.1	2	0.69	55.4	0.0	13.4
Italy	ITA21	QUEILE	2.9	2	0.69	44.6	0.0	13.4
Italy	ITA22	CASSAT	9.9	4	1.30	37.2	0.0	13.7
Italy	ITA22	OSTCAR	2.4	4	1.30	19.7	0.0	13.7
Italy	ITA22	QUECER	3.3	4	1.30	32.0	0.0	13.7
Italy	ITA22	QUEILE	1.8	4	1.30	11.1	0.0	13.7
Italy	ITA23	CASSAT	5.0	4	1.23	12.7	0.0	12.8
Italy	ITA23	QUECER	4.1	4	1.23	16.6	0.0	12.8
Italy	ITA23	QUEILE	4.2	4	1.23	20.7	0.0	12.8
Italy	ITA23	QUERCUS	9.2	4	1.23	50.1	0.0	12.8
Italy	ITA24	QUECER	2.4	2	0.62	69.1	0.0	13.2
Italy	ITA24	QUEILE	2.8	2	0.62	30.9	0.0	13.2
Italy	ITA25	CASSAT	4.8	3	0.89	19.6	0.0	13.4
Italy	ITA25	OSTCAR	0.3	3	0.89	64.8	0.0	13.4
Italy	ITA25	QUEILE	2.0	3	0.89	15.6	0.0	13.4
Italy	ITA26	CASSAT	9.5	3	1.08	24.2	0.0	13.2
Italy	ITA26	QUECER	6.8	3	1.08	40.1	0.0	13.2
Italy	ITA26	QUERCUS	8.8	3	1.08	35.7	0.0	13.2
Italy	ITA27	QUERCUS	11.2	1	0.25	93.2	0.0	13.2
Italy	ITA29	OSTCAR	1.1	3	0.93	12.2	0.0	13.6
Italy	ITA29	QUECER	6.1	3	0.93	61.0	0.0	13.6
Italy	ITA29	QUERCUS	6.8	3	0.93	26.5	0.0	13.6
Italy	ITA30	CASSAT	27.6	2	0.77	30.0	0.0	13.3
Italy	ITA30	OSTCAR	0.6	2	0.77	65.7	0.0	13.3
Italy	ITA31	OSTCAR	2.2	1	0.28	92.9	0.0	13.9
Italy	ITA32	CASSAT	7.1	4	1.27	46.8	0.0	12.4
Italy	ITA32	OSTCAR	0.4	4	1.27	20.8	0.0	12.4
Italy	ITA32	QUECER	0.7	4	1.27	16.8	0.0	12.4
Italy	ITA32	QUEILE	0.7	4	1.27	15.7	0.0	12.4
Italy	ITA33	CASSAT	18.2	4	1.32	40.3	0.0	12.4
Italy	ITA33	QUECER	0.8	4	1.32	23.0	0.0	12.4

country	id.plot	species	defoliation	richness	shannon	focal.prop	conifer.prop	temperature
Italy	ITA33	QUEILE	2.1	4	1.32	22.0	0.0	12.4
Italy	ITA33	QUERCUS	2.0	4	1.32	14.7	0.0	12.4
Italy	ITA34	CASSAT	18.5	5	1.55	17.7	0.0	12.4
Italy	ITA34	OSTCAR	3.3	5	1.55	12.8	0.0	12.4
Italy	ITA34	QUECER	2.3	5	1.55	13.8	0.0	12.4
Italy	ITA34	QUEILE	2.8	5	1.55	26.1	0.0	12.4
Italy	ITA34	QUERCUS	6.0	5	1.55	29.7	0.0	12.4
Italy	ITA35	QUECER	6.5	1	0.00	100.0	0.0	14.1
Italy	ITA36	CASSAT	14.0	3	0.91	14.6	0.0	13.2
Italy	ITA36	QUECER	9.4	3	0.91	18.3	0.0	13.2
Italy	ITA36	QUERCUS	6.8	3	0.91	66.3	0.0	13.2
Poland	POL01	PICABI	1.1	1	0.11	97.7	97.7	7.0
Poland	POL02	CARBET	9.5	1	0.40	89.6	4.4	6.9
Poland	POL03	PICABI	0.4	1	0.12	97.4	97.4	6.9
Poland	POL04	BETPEN	4.9	3	1.30	33.0	26.6	6.8
Poland	POL04	PICABI	0.6	3	1.30	26.6	26.6	6.8
Poland	POL04	QUERCUS	6.8	3	1.30	31.5	26.6	6.8
Poland	POL05	CARBET	13.9	2	0.69	51.5	0.0	6.9
Poland	POL05	QUERCUS	9.5	2	0.69	48.5	0.0	6.9
Poland	POL06	BETPEN	5.5	3	1.13	27.9	44.1	6.8
Poland	POL06	CARBET	6.8	3	1.13	26.6	44.1	6.8
Poland	POL06	PICABI	0.6	3	1.13	44.1	44.1	6.8
Poland	POL07	BETPEN	4.5	4	1.33	19.3	36.3	6.8
Poland	POL07	CARBET	12.7	4	1.33	14.9	36.3	6.8
Poland	POL07	PICABI	2.1	4	1.33	36.3	36.3	6.8
Poland	POL07	QUERCUS	4.8	4	1.33	29.4	36.3	6.8
Poland	POL08	BETPEN	2.8	2	0.69	51.0	0.0	6.8
Poland	POL08	CARBET	9.2	2	0.69	49.0	0.0	6.8
Poland	POL09	CARBET	14.3	2	0.78	31.0	64.7	6.8
Poland	POL09	PICABI	1.6	2	0.78	64.7	64.7	6.8
Poland	POL10	QUERCUS	20.4	1	0.55	81.8	2.9	6.8
Poland	POL11	BETPEN	2.5	1	0.56	75.2	0.0	6.8
Poland	POL12	CARBET	12.7	1	0.21	94.7	5.3	6.8
Poland	POL13	BETPEN	3.7	3	1.08	27.5	0.0	6.9
Poland	POL13	CARBET	25.0	3	1.08	43.1	0.0	6.9
Poland	POL13	QUERCUS	25.2	3	1.08	29.4	0.0	6.9
Poland	POL14	CARBET	30.7	4	1.30	13.7	70.7	6.9
Poland	POL14	PICABI	0.4	4	1.30	38.2	70.7	6.9
Poland	POL14	PINSYL	0.0	4	1.30	32.4	70.7	6.9
Poland	POL14	QUERCUS	6.1	4	1.30	15.7	70.7	6.9
Poland	POL15	CARBET	28.0	3	1.08	26.0	33.6	6.8
Poland	POL15	PICABI	2.2	3	1.08	33.6	33.6	6.8
Poland	POL15	QUERCUS	21.2	3	1.08	40.3	33.6	6.8
Poland	POL16	PICABI	0.5	2	0.85	45.5	45.5	6.8
Poland	POL16	QUERCUS	20.5	2	0.85	49.5	45.5	6.8

country	id.plot	species	defoliation	richness	shannon	focal.prop	conifer.prop	temperature
Poland	POL17	BETPEN	0.8	4	1.37	23.2	57.6	6.9
Poland	POL17	CARBET	5.2	4	1.37	19.2	57.6	6.9
Poland	POL17	PICABI	6.0	4	1.37	28.8	57.6	6.9
Poland	POL17	PINSYL	0.2	4	1.37	28.9	57.6	6.9
Poland	POL18	CARBET	6.8	3	1.15	14.2	46.7	6.9
Poland	POL18	PINSYL	0.0	3	1.15	46.7	46.7	6.9
Poland	POL18	QUERCUS	6.8	3	1.15	34.1	46.7	6.9
Poland	POL19	PICABI	0.2	2	0.77	56.5	98.0	6.9
Poland	POL19	PINSYL	0.0	2	0.77	41.5	98.0	6.9
Poland	POL20	CARBET	18.8	2	0.55	17.6	1.7	7.0
Poland	POL20	QUERCUS	14.4	2	0.55	80.7	1.7	7.0
Poland	POL21	PINSYL	0.0	1	0.34	91.8	91.8	6.9
Poland	POL22	PICABI	0.4	3	1.21	35.7	71.9	6.9
Poland	POL22	PINSYL	0.0	3	1.21	36.3	71.9	6.9
Poland	POL22	QUERCUS	5.9	3	1.21	24.2	71.9	6.9
Poland	POL23	CARBET	6.8	3	1.06	21.4	78.6	6.9
Poland	POL23	PICABI	0.2	3	1.06	42.5	78.6	6.9
Poland	POL23	PINSYL	0.0	3	1.06	36.1	78.6	6.9
Poland	POL24	CARBET	7.5	3	1.26	38.1	31.2	6.9
Poland	POL24	PINSYL	0.0	3	1.26	29.6	31.2	6.9
Poland	POL24	QUERCUS	6.8	3	1.26	27.4	31.2	6.9
Poland	POL25	CARBET	6.3	2	0.75	34.9	63.6	6.9
Poland	POL25	PINSYL	0.0	2	0.75	62.9	63.6	6.9
Poland	POL26	BETPEN	1.4	2	0.69	69.0	2.0	6.9
Poland	POL26	CARBET	18.3	2	0.69	29.0	2.0	6.9
Poland	POL27	CARBET	13.8	4	1.32	25.8	51.1	6.8
Poland	POL27	PICABI	0.0	4	1.32	38.3	51.1	6.8
Poland	POL27	PINSYL	0.0	4	1.32	12.9	51.1	6.8
Poland	POL27	QUERCUS	5.6	4	1.32	23.1	51.1	6.8
Poland	POL28	BETPEN	2.7	2	0.80	32.8	63.4	6.8
Poland	POL28	PICABI	0.5	2	0.80	63.4	63.4	6.8
Poland	POL29	BETPEN	2.4	3	1.27	15.3	53.5	6.8
Poland	POL29	PINSYL	0.0	3	1.27	51.4	53.5	6.8
Poland	POL29	QUERCUS	18.5	3	1.27	21.4	53.5	6.8
Poland	POL30	BETPEN	2.8	2	1.24	8.6	75.2	6.9
Poland	POL30	PICABI	2.2	2	1.24	40.3	75.2	6.9
Poland	POL30	PINSYL	0.0	2	1.24	34.9	75.2	6.9
Poland	POL31	BETPEN	2.8	4	1.29	16.7	24.5	6.9
Poland	POL31	CARBET	18.0	4	1.29	43.9	24.5	6.9
Poland	POL31	PICABI	0.0	4	1.29	24.5	24.5	6.9
Poland	POL31	QUERCUS	9.1	4	1.29	14.9	24.5	6.9
Poland	POL32	BETPEN	3.7	4	1.52	14.7	46.7	6.8
Poland	POL32	PICABI	2.9	4	1.52	23.3	46.7	6.8
Poland	POL32	PINSYL	0.0	4	1.52	23.4	46.7	6.8
Poland	POL32	QUERCUS	8.8	4	1.52	30.8	46.7	6.8

country	id.plot	species	defoliation	richness	shannon	focal.prop	conifer.prop	temperature
Poland	POL33	BETPEN	2.1	3	1.22	21.8	52.4	6.9
Poland	POL33	CARBET	22.9	3	1.22	25.8	52.4	6.9
Poland	POL33	PINSYL	0.0	3	1.22	45.5	52.4	6.9
Poland	POL34	BETPEN	4.1	4	1.36	29.8	18.0	7.0
Poland	POL34	CARBET	10.3	4	1.36	31.5	18.0	7.0
Poland	POL34	PINSYL	0.0	4	1.36	18.0	18.0	7.0
Poland	POL34	QUERCUS	7.4	4	1.36	20.6	18.0	7.0
Poland	POL35	BETPEN	4.1	5	1.57	15.7	46.8	6.8
Poland	POL35	CARBET	4.6	5	1.57	21.3	46.8	6.8
Poland	POL35	PICABI	0.2	5	1.57	31.0	46.8	6.8
Poland	POL35	PINSYL	0.0	5	1.57	15.8	46.8	6.8
Poland	POL35	QUERCUS	6.4	5	1.57	16.2	46.8	6.8
Poland	POL36	BETPEN	3.3	3	1.25	44.8	10.0	6.9
Poland	POL36	QUERCUS	41.2	3	1.25	28.3	10.0	6.9
Poland	POL37	BETPEN	3.1	5	1.53	14.1	57.4	6.9
Poland	POL37	CARBET	30.6	5	1.53	12.0	57.4	6.9
Poland	POL37	PICABI	0.1	5	1.53	35.1	57.4	6.9
Poland	POL37	PINSYL	0.0	5	1.53	22.3	57.4	6.9
Poland	POL37	QUERCUS	6.8	5	1.53	16.5	57.4	6.9
Poland	POL38	BETPEN	3.9	2	0.91	17.5	67.4	7.0
Poland	POL38	PINSYL	0.0	2	0.91	67.4	67.4	7.0
Poland	POL39	BETPEN	2.8	4	1.44	24.7	23.6	7.0
Poland	POL39	CARBET	13.9	4	1.44	32.4	23.6	7.0
Poland	POL39	PINSYL	0.0	4	1.44	21.4	23.6	7.0
Poland	POL39	QUERCUS	6.8	4	1.44	19.3	23.6	7.0
Poland	POL40	PINSYL	0.0	1	0.39	88.3	89.2	6.9
Poland	POL41	BETPEN	3.9	4	1.31	40.9	34.2	6.7
Poland	POL41	PICABI	2.3	4	1.31	23.0	34.2	6.7
Poland	POL41	PINSYL	0.0	4	1.31	11.2	34.2	6.7
Poland	POL41	QUERCUS	6.8	4	1.31	24.7	34.2	6.7
Poland	POL42	BETPEN	0.8	4	1.38	23.8	53.7	6.8
Poland	POL42	CARBET	7.2	4	1.38	22.5	53.7	6.8
Poland	POL42	PICABI	0.4	4	1.38	30.8	53.7	6.8
Poland	POL42	PINSYL	0.0	4	1.38	22.9	53.7	6.8
Poland	POL43	PICABI	0.7	3	1.19	31.2	74.3	7.0
Poland	POL43	PINSYL	0.2	3	1.19	43.1	74.3	7.0
Poland	POL43	QUERCUS	6.8	3	1.19	21.7	74.3	7.0
Romania	ROM01	PICABI	0.0	1	0.16	96.9	98.9	5.8
Romania	ROM02	PICABI	0.0	1	0.00	100.0	100.0	5.8
Romania	ROM03	ACEPSE	9.2	3	1.14	21.9	26.2	5.8
Romania	ROM03	FAGSYL	17.4	3	1.14	51.9	26.2	5.8
Romania	ROM03	PICABI	0.0	3	1.14	22.1	26.2	5.8
Romania	ROM05	ABIALB	0.6	2	0.73	63.1	98.2	5.6
Romania	ROM05	PICABI	0.6	2	0.73	35.1	98.2	5.6
Romania	ROM06	ACEPSE	6.1	2	0.97	49.6	8.0	5.6

country	id.plot	species	defoliation	richness	shannon	focal.prop	conifer.prop	temperature
Romania	ROM06	FAGSYL	6.5	2	0.97	42.3	8.0	5.6
Romania	ROM07	ABIALB	0.2	1	0.12	97.5	97.5	5.2
Romania	ROM08	ABIALB	0.9	4	1.33	29.2	44.5	5.4
Romania	ROM08	ACEPSE	5.2	4	1.33	18.5	44.5	5.4
Romania	ROM08	FAGSYL	5.9	4	1.33	37.0	44.5	5.4
Romania	ROM08	PICABI	0.5	4	1.33	15.4	44.5	5.4
Romania	ROM09	FAGSYL	13.4	2	0.86	51.6	45.7	5.4
Romania	ROM09	PICABI	0.0	2	0.86	44.2	45.7	5.4
Romania	ROM10	ABIALB	0.0	4	1.14	23.3	78.5	5.4
Romania	ROM10	ACEPSE	6.8	4	1.14	13.2	78.5	5.4
Romania	ROM10	FAGSYL	6.8	4	1.14	8.4	78.5	5.4
Romania	ROM10	PICABI	0.0	4	1.14	55.2	78.5	5.4
Romania	ROM11	ABIALB	0.4	3	1.13	22.7	69.7	5.8
Romania	ROM11	FAGSYL	10.3	3	1.13	28.4	69.7	5.8
Romania	ROM11	PICABI	0.5	3	1.13	47.0	69.7	5.8
Romania	ROM12	FAGSYL	13.9	2	0.69	50.9	49.1	5.7
Romania	ROM12	PICABI	0.0	2	0.69	49.1	49.1	5.7
Romania	ROM13	FAGSYL	15.1	1	0.00	100.0	0.0	5.7
Romania	ROM14	ACEPSE	6.8	2	0.66	63.0	0.0	5.2
Romania	ROM14	FAGSYL	9.9	2	0.66	37.0	0.0	5.2
Romania	ROM15	ACEPSE	7.8	1	0.29	93.2	6.8	5.2
Romania	ROM16	ACEPSE	6.8	1	0.34	91.8	3.3	5.2
Romania	ROM17	ABIALB	0.2	2	0.64	21.5	97.1	4.6
Romania	ROM17	PICABI	0.4	2	0.64	75.6	97.1	4.6
Romania	ROM18	ABIALB	0.6	3	1.09	37.0	72.2	4.6
Romania	ROM18	FAGSYL	10.1	3	1.09	27.8	72.2	4.6
Romania	ROM18	PICABI	0.2	3	1.09	35.2	72.2	4.6
Romania	ROM19	ABIALB	2.7	2	0.76	22.8	29.3	5.7
Romania	ROM19	FAGSYL	7.7	2	0.76	70.7	29.3	5.7
Romania	ROM20	ABIALB	0.4	3	1.08	25.5	26.6	5.7
Romania	ROM20	ACEPSE	5.2	3	1.08	22.9	26.6	5.7
Romania	ROM20	FAGSYL	19.5	3	1.08	50.5	26.6	5.7
Romania	ROM21	ACEPSE	6.3	3	1.07	52.3	18.0	5.7
Romania	ROM21	FAGSYL	13.1	3	1.07	29.6	18.0	5.7
Romania	ROM21	PICABI	0.6	3	1.07	16.0	18.0	5.7
Romania	ROM22	ABIALB	0.4	3	1.06	29.1	74.9	6.2
Romania	ROM22	FAGSYL	13.4	3	1.06	25.1	74.9	6.2
Romania	ROM22	PICABI	0.4	3	1.06	45.8	74.9	6.2
Romania	ROM23	ABIALB	0.3	1	0.06	99.0	99.0	5.6
Romania	ROM24	ABIALB	0.2	2	0.67	59.5	59.5	5.6
Romania	ROM24	FAGSYL	6.8	2	0.67	40.5	59.5	5.6
Romania	ROM25	ABIALB	0.0	3	1.14	14.8	18.6	4.6
Romania	ROM25	ACEPSE	4.8	3	1.14	41.0	18.6	4.6
Romania	ROM25	FAGSYL	5.6	3	1.14	40.4	18.6	4.6
Romania	ROM26	FAGSYL	12.8	1	0.13	97.4	2.6	5.9

country	id.plot	species	defoliation	richness	shannon	focal.prop	conifer.prop	temperature
Romania	ROM27	ACEPSE	6.8	2	1.02	53.8	42.0	6.3
Romania	ROM27	PICABI	0.0	2	1.02	35.1	42.0	6.3
Romania	ROM28	ABIALB	0.0	2	0.79	69.6	78.0	6.5
Romania	ROM28	FAGSYL	12.9	2	0.79	22.0	78.0	6.5
Romania	ROM29	ABIALB	0.2	4	1.37	23.5	43.8	5.2
Romania	ROM29	ACEPSE	5.9	4	1.37	24.7	43.8	5.2
Romania	ROM29	FAGSYL	8.2	4	1.37	31.4	43.8	5.2
Romania	ROM29	PICABI	0.2	4	1.37	20.3	43.8	5.2
Spain	SPA01	PINSYL	0.1	2	0.85	30.9	38.2	9.7
Spain	SPA01	QUEFAG	15.3	2	0.85	61.8	38.2	9.7
Spain	SPA02	PINNIG	1.7	3	1.13	42.7	71.4	9.7
Spain	SPA02	PINSYL	0.2	3	1.13	28.7	71.4	9.7
Spain	SPA02	QUEFAG	9.3	3	1.13	27.5	71.4	9.7
Spain	SPA03	PINNIG	0.0	2	0.62	68.6	68.6	9.7
Spain	SPA03	QUEFAG	12.0	2	0.62	31.4	68.6	9.7
Spain	SPA04	PINSYL	0.0	2	0.62	69.2	69.2	9.7
Spain	SPA04	QUEFAG	8.9	2	0.62	30.8	69.2	9.7
Spain	SPA05	PINSYL	0.1	2	0.60	29.1	29.1	9.7
Spain	SPA05	QUEFAG	5.5	2	0.60	70.9	29.1	9.7
Spain	SPA06	PINNIG	0.0	2	0.66	63.2	63.2	9.7
Spain	SPA06	QUEFAG	12.8	2	0.66	36.8	63.2	9.7
Spain	SPA07	PINNIG	0.0	3	0.86	24.8	89.5	9.7
Spain	SPA07	PINSYL	0.0	3	0.86	64.7	89.5	9.7
Spain	SPA07	QUEFAG	6.8	3	0.86	10.5	89.5	9.7
Spain	SPA08	PINNIG	0.2	2	0.69	50.9	50.9	9.6
Spain	SPA08	QUEFAG	12.9	2	0.69	49.1	50.9	9.6
Spain	SPA09	QUEFAG	19.2	1	0.00	100.0	0.0	9.6
Spain	SPA10	QUEFAG	6.3	1	0.00	100.0	0.0	9.6
Spain	SPA11	QUEFAG	6.2	1	0.00	100.0	0.0	9.9
Spain	SPA12	PINNIG	0.0	1	0.00	100.0	100.0	10.0
Spain	SPA13	PINNIG	1.6	3	1.04	26.6	26.6	10.8
Spain	SPA13	QUEFAG	1.9	3	1.04	49.5	26.6	10.8
Spain	SPA13	QUEILE	0.8	3	1.04	23.9	26.6	10.8
Spain	SPA14	PINNIG	0.0	2	0.77	66.5	66.5	10.8
Spain	SPA14	QUEILE	4.8	2	0.77	28.7	66.5	10.8
Spain	SPA15	PINNIG	0.1	1	0.09	98.1	98.1	10.8
Spain	SPA16	PINNIG	0.0	2	0.75	67.6	67.6	10.8
Spain	SPA16	QUEILE	0.8	2	0.75	28.5	67.6	10.8
Spain	SPA17	PINNIG	0.1	1	0.02	99.7	99.7	10.8
Spain	SPA18	PINSYL	0.1	1	0.00	100.0	100.0	9.1
Spain	SPA19	PINSYL	0.0	1	0.00	100.0	100.0	9.2
Spain	SPA20	PINSYL	0.0	1	0.00	100.0	100.0	9.1
Spain	SPA21	PINNIG	0.2	2	0.68	57.4	100.0	9.0
Spain	SPA21	PINSYL	0.0	2	0.68	42.6	100.0	9.0
Spain	SPA22	PINNIG	0.1	2	0.69	44.7	100.0	9.0

country	id.plot	species	defoliation	richness	shannon	focal.prop	conifer.prop	temperature
Spain	SPA22	PINSYL	0.0	2	0.69	55.3	100.0	9.0
Spain	SPA23	PINNIG	0.9	2	0.84	56.9	94.9	9.1
Spain	SPA23	PINSYL	0.1	2	0.84	38.0	94.9	9.1
Spain	SPA24	PINNIG	0.4	4	1.35	25.7	54.5	9.1
Spain	SPA24	PINSYL	0.1	4	1.35	28.8	54.5	9.1
Spain	SPA24	QUEFAG	5.9	4	1.35	30.8	54.5	9.1
Spain	SPA24	QUEILE	0.6	4	1.35	14.7	54.5	9.1
Spain	SPA25	PINNIG	0.0	3	1.28	42.6	71.1	9.1
Spain	SPA25	PINSYL	0.0	3	1.28	28.6	71.1	9.1
Spain	SPA25	QUEFAG	5.6	3	1.28	17.2	71.1	9.1
Spain	SPA26	PINNIG	0.4	4	1.35	34.2	52.4	9.1
Spain	SPA26	PINSYL	0.1	4	1.35	18.2	52.4	9.1
Spain	SPA26	QUEFAG	3.6	4	1.35	27.8	52.4	9.1
Spain	SPA26	QUEILE	0.3	4	1.35	19.9	52.4	9.1
Spain	SPA27	PINNIG	1.2	3	1.09	39.0	39.0	9.3
Spain	SPA27	QUEFAG	8.0	3	1.09	30.4	39.0	9.3
Spain	SPA27	QUEILE	0.2	3	1.09	30.6	39.0	9.3
Spain	SPA28	QUEFAG	14.2	2	0.56	24.7	0.0	9.4
Spain	SPA28	QUEILE	2.4	2	0.56	75.3	0.0	9.4
Spain	SPA29	PINNIG	3.2	4	1.23	47.5	74.2	9.4
Spain	SPA29	PINSYL	0.1	4	1.23	26.7	74.2	9.4
Spain	SPA29	QUEFAG	3.6	4	1.23	12.8	74.2	9.4
Spain	SPA29	QUEILE	0.6	4	1.23	13.0	74.2	9.4
Spain	SPA30	QUEFAG	14.9	2	0.68	41.7	0.0	9.4
Spain	SPA30	QUEILE	0.5	2	0.68	58.3	0.0	9.4
Spain	SPA31	QUEFAG	8.8	2	0.61	69.5	0.0	9.3
Spain	SPA31	QUEILE	0.4	2	0.61	30.5	0.0	9.3
Spain	SPA32	QUEILE	1.7	1	0.00	100.0	0.0	10.3
Spain	SPA33	QUEILE	1.2	1	0.00	100.0	0.0	10.3
Spain	SPA34	QUEFAG	16.8	1	0.46	17.4	0.0	10.7
Spain	SPA34	QUEILE	2.5	1	0.46	82.6	0.0	10.7
Spain	SPA35	PINNIG	0.8	3	0.95	51.0	51.0	9.9
Spain	SPA35	QUEFAG	5.0	3	0.95	10.3	51.0	9.9
Spain	SPA35	QUEILE	1.8	3	0.95	38.7	51.0	9.9
Spain	SPA36	PINNIG	0.2	2	0.69	55.9	55.9	10.2
Spain	SPA36	QUEILE	1.5	2	0.69	44.1	55.9	10.2
